# Chronic delta-9-tetrahydrocannabinol (THC) treatment counteracts SIV-induced modulation of proinflammatory microRNA cargo in basal ganglia-derived extracellular vesicles

**DOI:** 10.1186/s12974-022-02586-9

**Published:** 2022-09-12

**Authors:** Hussein Kaddour, Marina McDew-White, Miguel M. Madeira, Malik A. Tranquille, Stella E. Tsirka, Mahesh Mohan, Chioma M. Okeoma

**Affiliations:** 1 Department of Pharmacology, Stony Brook University Renaissance School of Medicine, Stony Brook, NY 11794-8651 USA; 2 Present Address: Regeneron Pharmaceuticals, Inc., Tarrytown, NY 10591 USA; 3 Host Pathogen Interaction Program, Southwest National Primate Research Center, Texas Biomedical Research Institute, San Antonio, TX 78227-5302 USA; 4 Department of Pathology, Microbiology, and Immunology, New York Medical College, Valhalla, NY 10595-1524 USA

**Keywords:** Basal ganglia, Astrocytes, CX3CR1, Extracellular vesicles, THC, Neuroinflammation

## Abstract

**Background:**

Early invasion of the central nervous system (CNS) by human immunodeficiency virus (HIV) (Gray et al. in Brain Pathol 6:1–15, 1996; An et al. in Ann Neurol 40:611–6172, 1996), results in neuroinflammation, potentially through extracellular vesicles (EVs) and their micro RNAs (miRNA) cargoes (Sharma et al. in FASEB J 32:5174–5185, 2018; Hu et al. in Cell Death Dis 3:e381, 2012). Although the basal ganglia (BG) is a major target and reservoir of HIV in the CNS (Chaganti et al. in Aids 33:1843–1852, 2019; Mintzopoulos et al. in Magn Reson Med 81:2896–2904, 2019), whether BG produces EVs and the effect of HIV and/or the phytocannabinoid–delta-9-tetrahydrocannabinol (THC) on BG-EVs and HIV neuropathogenesis remain unknown.

**Methods:**

We used the simian immunodeficiency virus (SIV) model of HIV and THC treatment in rhesus macaques (Molina et al. in AIDS Res Hum Retroviruses 27:585–592, 2011) to demonstrate for the first time that BG contains EVs (BG-EVs), and that BG-EVs cargo and function are modulated by SIV and THC. We also used primary astrocytes from the brains of wild type (WT) and CX3CR1^+/GFP^ mice to investigate the significance of BG-EVs in CNS cells.

**Results:**

Significant changes in BG-EV-associated miRNA specific to SIV infection and THC treatment were observed. BG-EVs from SIV-infected rhesus macaques (SIV EVs) contained 11 significantly downregulated miRNAs. Remarkably, intervention with THC led to significant upregulation of 37 miRNAs in BG-EVs (SIV–THC EVs). Most of these miRNAs are predicted to regulate pathways related to inflammation/immune regulation, TLR signaling, Neurotrophin TRK receptor signaling, and cell death/response. BG-EVs activated WT and CX3CR1^+/GFP^ astrocytes and altered the expression of CD40, TNFα, MMP-2, and MMP-2 gene products in primary mouse astrocytes in an EV and CX3CR1 dependent manners.

**Conclusions:**

Our findings reveal a role for BG-EVs as a vehicle with potential to disseminate HIV- and THC-induced changes within the CNS.

**Supplementary Information:**

The online version contains supplementary material available at 10.1186/s12974-022-02586-9.

## Background

The advent of antiretroviral therapy (ART) has increased the lifespans of people living with HIV (PLWH) [8]. However, a significantly high percentage of PLWH remain comorbid with drug abuse, (cocaine and marijuana [9–11]) leading to rapid disease progression [12–17], immune activation, or protection from immune activation in cases of dual drug use (marijuana and cocaine) [9]. Although HIV-induced neuroinflammation may drive HIV-associated neurocognitive disorder (HAND), the mechanisms by which cannabinoids, such as THC, a component of marijuana modulates HIV-induced neuroinflammation are not completely understood.

Investigating the longitudinal effects of HIV and THC on the brain is difficult to conduct in humans, because humans are polydrug users, cigarette smokers, and alcohol consumers. Humans also have variability in patterns and lengths of drug, alcohol, and cigarette usage. These extrinsic factors make studies with human subjects’ complex, and data associated with such studies correlational. Animal models, such as the SIV-infected rhesus macaque (SIV/RM) model [18, 19] of HIV provides a valuable animal model/approach and more controlled environment to study HIV-induced neuroinflammation, the response to long-term treatment with THC, and the effects of HIV/SIV alone or SIV and THC on EV cargo composition and function.

Previously, we showed that the anti-inflammatory effects of long-term low dose THC was associated with its ability to stimulate the release of bioactive blood-derived extracellular (BEVs) [18] that induced divergent actin cytoskeletal and signaling cues in SIV-infected RMs [18]. Whether or not THC is capable of reversing neuroinflammatory effects of HIV/SIV is unknown. In addition to the effects of HIV proteins and RNA on persistent inflammation/immune activation, EVs play multifaceted roles in the function/dysfunction of HIV target cells, such as monocytes, microglial, astroglial and non-target cells, including neurons. Broadly defined, EVs released by many cell types [20–32] encompassing exosomes, microvesicles, and apoptotic bodies carry bioinformation (proteins, DNA, diverse RNA profiles) and regulate intercellular/organ communications [24, 29, 31]. EVs carry markers of the producer cells and if the producer cells are healthy or diseased, EVs will carry markers corresponding to their state [33, 34]. Thus, EVs are important in research [26, 33, 35–37] and drug discovery/therapeutics [38].

The cargo of EVs, including RNA mediate dynamic intercellular crosstalk. EV-associated RNA (exRNA) consists of diverse RNA biotypes that are incorporated into or associated with various circulating carriers, including lipoproteins [39, 40], EVs [41, 42] and extracellular or membraneless condensates (MCs) [42]. EV-associated exRNA include several classes of long and small RNAs but not limited to miRNAs [32]. Through association with EVs, miRNAs are protected from degradation by RNAses [43].

EVs traverse the blood brain barrier (BBB) and have been shown to regulate the availability of neuroprotective factors [4]. Emerging evidence indicate that brain-derived EVs are linked to neurogenesis, neural development, synaptic communication, nerve regeneration, and neuroinflammation [44–47]. EVs are used to establish molecular signatures associated with drug abuse in HIV-infected individuals [4, 18, 48, 49], and as such, may serve as intercellular conveyors of bioactive molecules within the CNS. Indeed, brain-derived EVs have been purified from cultured neurons, oligodendrocytes, microglia, astrocytes, and cerebrospinal fluid (CSF) [50–52]. However, there are no available studies describing the properties and functions of EVs from the basal ganglia (BG), a series of interconnected subcortical nuclei and a major target/reservoir of HIV in the CNS [5, 6]. BG dysfunction is a hallmark of HIV infection and cognitive impairment in PLWH with neuronal death in the BG [53]. Moreover, HIV targets the BG leading to loss of dopaminergic neurons [54]. A prior study showed an increase in miR-29b in the BG of morphine-dependent SIV-infected RMs compared with controls [4].

In this study, we demonstrate that BG contains bioactive EVs. A significant number of miRNAs are significantly decreased in the EVs isolated from BG of SIV-infected RMs. We further demonstrate that low-dose chronic THC treatment counteracts the suppressive effects of SIV on BG-derived EV miRNA repertoire, and conversely restores the levels of all of the suppressed miRNAs. Furthermore, BG-EVs internalized by astrocytes alter astrocyte activation and gene expression profiles in an EV and CX3CR1 dependent manner.

## Methods

### Macaques and viruses

A total of nine age and weight-matched Mamu-A0*1^−^/B08^−^/B17^−^ specific-pathogen-free (free of SIV, D retrovirus, STLV and Herpes B) male Indian rhesus macaques were randomly assigned to three experimental groups. One group (*n* = 3; Group 1) received twice daily injections of vehicle (VEH/SIV) (1:1:18 of emulphor:alcohol:saline) and second (*n* = 3; Group 2) received twice-daily injections of Δ^9^-THC (THC/SIV) beginning 4 weeks prior to SIV infection until 6 month post-SIV infection [55]. Group 3 (*n* = 3) macaques served as uninfected controls (Table 1). THC (NIDA/NIH) was prepared as an emulsion using alcohol, emulphor, and saline (1:1:18) as vehicle before use. Chronic administration of VEH (Group 1) or Δ^9^-THC (Group 2) was initiated 4 weeks before SIV infection at 0.18 mg/kg as used in previous studies [7, 55–57]. This dose of Δ^9^-THC was found to eliminate responding in a complex operant behavioral task in almost all animals [57]. Groups 1 and 2 macaques were infected intravenously with 100 TCID_50_ dose of the CCR5 tropic SIVmac251. Beginning the day of SIV infection, the THC dose was increased for each subject to 0.32 mg/kg, over a period of approximately 2 weeks when responding was no longer affected by 0.18 mg/kg on a daily basis (i.e., tolerance developed), and maintained for the duration of the study. The optimization of the THC dosing in RMs accounts for the development of tolerance during the initial period of administration. Because in previously published studies [7, 57] this dose of THC showed protection, the same dose was used in this study. At necropsy, BG tissues were collected in RNAlater (Thermo Fisher Scientific) and Z-fix for total RNA extraction and embedding in paraffin blocks. SIV levels in plasma and BG were quantified using the TaqMan One-Step Real-time RT-qPCR assay that targeted the LTR gene [55, 56].

**Table 1:** Animal IDs, SIV inoculum, duration of infection, viral loads, and brain histopathology in vehicle or delta-9-tetrahydrocannabinol (Δ^9^-THC) treated chronic SIV-infected and uninfected rhesus macaques

Animal ID	SIV Inoculum	Duration of Infection	Plasma viral loads 10^6^/mL	Brain viral loads 10^6^/mg RNA	Brain Histopathology	Opportunistic Infections
Chronic SIV-Infected and Vehicle treated (Group 1)						
IV95	SIVmac251	180	0.02	2.0	ND	ND
JD66	SIVmac251	180	0.04	0.2	ND	ND
JR36	SIVmac251	180	0.5	0.2	ND	ND
Chronic SIV-Infected and Δ^9^-THC treated (Group 2)						
JI45	SIVmac251	180	3	0.01	ND	ND
JC85	SIVmac251	180	0.02	0.09	ND	ND
IV90	SIVmac251	180	0.02	0.06	ND	ND
Uninfected Controls (Group 3)						
IR97	NA	NA	NA	NA	NA	NA
IT18	NA	NA	NA	NA	NA	NA
GT18	NA	NA	NA	NA	NA	NA

*NA* not applicable, *ND* none detected

### BG-EV purification and characterization

The schematic and workflow for isolation of basal ganglia EVs is shown in Additional file 1: Fig. S1. Briefly, small chunks of RNALater-stored BG tissues, ranging from 35 to 118 mg, were finely chopped and digested with collagenase III. Samples were clarified and supernatant was purified on a 20 × 0.5 cm Sephadex G-50 size exclusion column, using a particle purification liquid chromatography (PPLC) system as previously described [42]. Fifty fractions of 200 µL were collected, and the 3D UV–Vis (230–650 nm) fractionation profiles were recorded. A no-tissue collagenase control was used as background. After background subtraction and PPLC analysis for particle size and concentration, EV-containing fractions were pooled and stored in small aliquots at − 80 °C. For further characterization, EVs were diluted in 0.1X PBS (1/1000). Zeta potential (ζ-potential) measurements were acquired using nanoparticle tracking analysis (ZetaView) as described previously [58].

### Energy dispersive X-ray transmission electron microscope with immunogold-labelling (TEM–EDX-IL)

Equal volumes of BG-EVs from each group were pooled (*n* = 4). 10 µL were spotted onto TEM grids. Specimens were incubated with anti-CD9 at 4 °C overnight. Following washing, samples were incubated with 10 nm gold-conjugated anti-mouse IgG for 1 h, washed, and then followed by a post-stain with uranyl acetate (1%). Specimens were characterized using TEM.

### BG-EV RNA isolation

2 mL of PPLC-purified BG-EVs (equivalent of 9.6 × 10^11^ to 5.7 × 10^12^ particles or 254–984 µg of EV proteins) were concentrated under reduced pressure at low temperature, and the total RNA was isolated using miRNeasy serum/plasma kit, per manufacturer’s protocol. RNA was eluted and the eluate was measured using a NanoDrop 1000.

### Small RNA-Seq

Libraries were prepared using 25 ng of RNA and 20 cycles of PCR following the manufacturer’s recommendations. The libraries were pooled to equal nanomolarity concentrations and then purified and size selected using Pippin Prep (Sage Biosciences, Beverly, MA, USA). The library pool was profiled using a TapeStation (Agilent Technologies, USA) and Qubit (ThermoFisher) before sequencing on the NextSeq 550 (Illumina, San Diego, CA, USA). Sequencing was performed with single 75 bp reads.

### Bioinformatics

sRNA-Seq data were processed for filtering, trimming, and QC analyses before generating count matrices. After adapters are trimmed, reads are filtered based on length (5 bp, 15 bp). Filtering reads shorter than 5 bp determines RNA degradation. 15 bp is the minimum length for meaningful alignments. Count matrices were obtained using trimmed reads (minimum length 15) by alignment to the *Macaca mulatta* genome and the *Macaca mulatta* data set miRBase (miRNA database). Raw miR Counts are provided in Additional file 2: Table S1.

### Mouse model

All animal procedures were approved by the Institutional Animal Care and Use Committee (IACUC; covered by Animal welfare assurance No A3011-0), SUNY Stony Brook, School of Medicine and conducted in accordance with National Institutes of Health “Guide for the Care and Use of Laboratory Animals” guidelines. Experiments were performed using adult (1–3 days) male mice [C57BL/6 J (wt) and CX3CR1-GFP (Jackson Labs, 005582 model B6.129P-Cx3cr1tm1Litt/J)9]. The mouse lines were backcrossed onto a C57BL/6 J background, bred in-house, and genotyped by PCR. The brains from these mouse strains were used for preparation of primary astrocyte cultures.

### Generation of primary cortical astrocytes

Primary astrocytes were isolated from P1–P3 mouse pups [59, 60]. Briefly, T-75 flasks were coated with Poly-d-Lysine for 1 h at 37 °C. Flasks were washed, filled with DMEM 10% FBS and placed at 37 °C, 5% CO_2_. Brains were dissected, the cerebellum removed, and the Cortices placed on ice in 1X HBSS to slow metabolic function. Four cortices were then placed in one mL of papain solution for 15 min at 37 °C for chemical dissociation, with a brief mechanical dissociation through a p1000 pipette tip. After additional incubation at 37 °C for 15 min, p1000 pipette dissociation, a p200 pipette was used to further digest the tissue into a homogenous single-cell suspension. Papain activity was neutralized using 20 mL of DMEM 10% FBS media and the solution spun at 3000 RPM for 10 min. The pellet was resuspended in 1 mL DMEM 10% FBS, placed into T-75 flask containing 37 °C media. On days 3, 6, and 9 of culture, a portion of 10 mL of media were removed and replaced with equivalent volume of fresh media. On day 10 of culture, microglia were detached and removed. On day 12 of culture, OPCs were detached (in a 16–18 h shake at 300 RPM) and removed. After removal of the OPCs, the remaining cells are astrocytes and were trypsinized and plated in a 10 cm dish.

### BG-EV internalization

PBS control or BG-EVs were stained with SYTO™ RNASelect™ Green Fluorescent cell stain and purified using Exosome Spin Columns. Labelled BG-EVs were added to cells for kinetic imaging using Lionheart FX automated scope.

###  Immunofluoresence of activation markers

10,000 astrocytes were treated with PBS (vehicle) or 100 µg/mL of pooled BG-EVs (*n* = 4, 25 µg/sample) for 24 h. Cells were imaged immediately after treatment and at 24 h post-treatment. Subsequently, cells were fixed, permeabilized, incubated with anti-GFAP, and detected with appropriate fluorescently conjugated secondary antibody. Cells were finally stained with DAPI and imaged again using a confocal microscope (Leica Sp8-x) or an automated scope (Lionheart FX). Briefly, nine fields of views per well and three wells per condition were recorded. Images were then pre-processed, deconvoluted, and stitched, and cells were identified using DAPI channel. A secondary mask was then applied in which Texas red MFI (representing GFAP) was calculated. For the WT: PBS: 1062 cells, Control EVs: 972 cells, VEH/SIV EVs: 817 cells, THC/SIV EVs: 1039 cells. For CX3CR1^+/GFP^: PBS: 591 cells, Control EVs: 714 cells, VEH/SIV EVs: 1001 cells, THC/SIV EVs: 828 cells.

### Real-time quantitative PCR (RT-qPCR)

250,000 astrocytes plated overnight were treated with PBS or with 100 µg/mL of pooled BG-EVs (*n* = 4, 25 µg/sample) for 24 h. RNA was extracted from cells and used for cDNA synthesis. RT-qPCR was performed using a 7500 FAST machine and Power Track SYBR Green master mix.

For validation of the let-7 family of miRNA, we used the Thermo Fisher Scientific mml-let-7a-5p (Assay ID: 000377) and mml-let-7c-5p (Assay ID: 000379) TaqMan PCR specific assays, following manufacturer’s instructions.

### Cell viability analysis

Percentage of viable cells was inferred by quantitation of cellular ATP, measured using CellTiter-Glo Luminescent viability assay [61, 62].

### Statistical analysis

Differential expression data were generated using Graphpad Prism. The significance cutoff was set to fold change (FC) > 1.5 or < − 1.5 and a *p* value < 0.05. Ordinary one-way ANOVA multiple comparison test or two-way ANOVA test with (Dunnett’s or Tukey’s corrections were used to assess statistical differences. When stated, unpaired *T* test with Welch’s correction was also used. Details of specific statistics are presented in each figure legend, where the *p* values are listed.

## Results

### Assessment of plasma and BG viral loads

Viral loads were generally higher in BG of VEH/SIV compared to THC/SIV rhesus macaques (Table 1). However, the differences did not reach statistical significance (*p* = 0.100).

### SIV infection and long-term low dose THC treatment do not alter BG-EV physicochemical properties

**Fig. 1:**
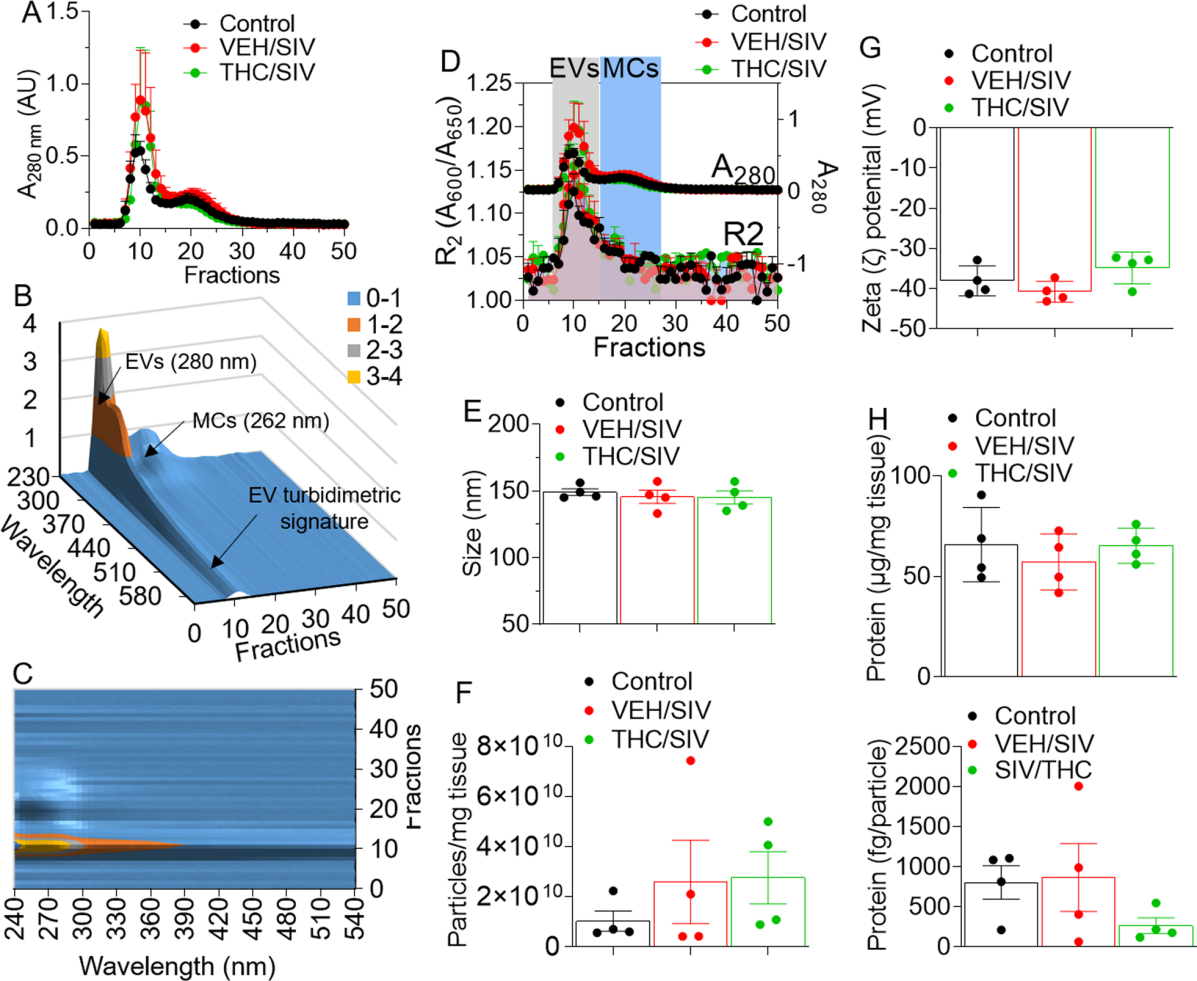
Physicochemical characterization of BG-EVs. **A** Absorbance at 280 nm of PPLC-isolated collagenase-digested BG eluates from VEH, SIV and THC/SIV groups (*n* = 4/group). **B** 3D-surface plot featuring the EV-turbidimetric signature. **C** Contour representation highlighting the non-membranous molecular condensates (MCs) whose UV-peak blue-shifted to 262 nm. **D** R2 index detected in fractions 7–16, confirming the presence of EVs in these fractions, whereas fractions 19–28 contained the MCs. **E, F** PPLC-derived particle size (**E**) and concentration (**F**). **G** Zeta (ζ)-potential of BG-EVs as measured by NTA (ZetaView). **H** Quantification of EV-associated total protein as measured by the Bradford assay (top) and assessment of the protein weight per EV particle (Bottom). *N* = 4 per group. Error bars represent standard error of the mean. No statistical differences were noted

Clarified supernatant from BG digest is a mixture of collagenase, EVs, and non-EVs. We used a Particle Purification Liquid Chromatography (PPLC) system [42] to (1) gain insight into the spectra of BG digest from the three experimental groups (uninfected controls, VEH/SIV and THC/SIV), and (2) identify and collect pure EVs devoid of other factors, such as non-EV membraneless condensates that often times co-purify with EVs [42]. Schematic for BG-EV isolation workflow is shown in Additional file 1: Fig. S1. The elution profiles of BG digest from uninfected controls, VEH/SIV, and THC/SIV groups are similar (Fig. 1A). 3D UV–Vis measurements (fraction/wavelength/intensity) showing 3D-surface plot of EV fractions (Fig. 1B) and MC UV-peak that blue-shifted to 262 nm (Fig. 1C) were generated using PPLC analytics. Qualitative turbidity indices in the visible range, defined as ${R}_{1}={A}_{400}/{A}_{600}$ and ${\mathrm{R}}_{2}={\mathrm{A}}_{600}/{\mathrm{A}}_{650}$ was used for further identification of the different fractions, where R_2_ index detected in fractions 7–16, indicate the presence of EVs, while fractions 19–28 contain MCs (Fig. 1D). As indicated in Fig. 1E, the sizes (133–157 nm) of BG-EVs from uninfected controls, VEH/SIV, and THC/SIV groups are not different. In addition, BG-EV concentration per mg of tissue was similar for all groups (Fig. 1F), but the ζ-potential of BG-EV membrane showed that BG-EVs bear highly negative surface charge (Fig. 1G). Furthermore, there were no differences in the total protein concentrations of BG-EVs in all three groups (Fig. 1H).

### SIV infection and long-term low dose THC treatment do not alter ultrastructural, elemental, and surface cargo of BG-EVs and BG MCs

**Fig. 2:**
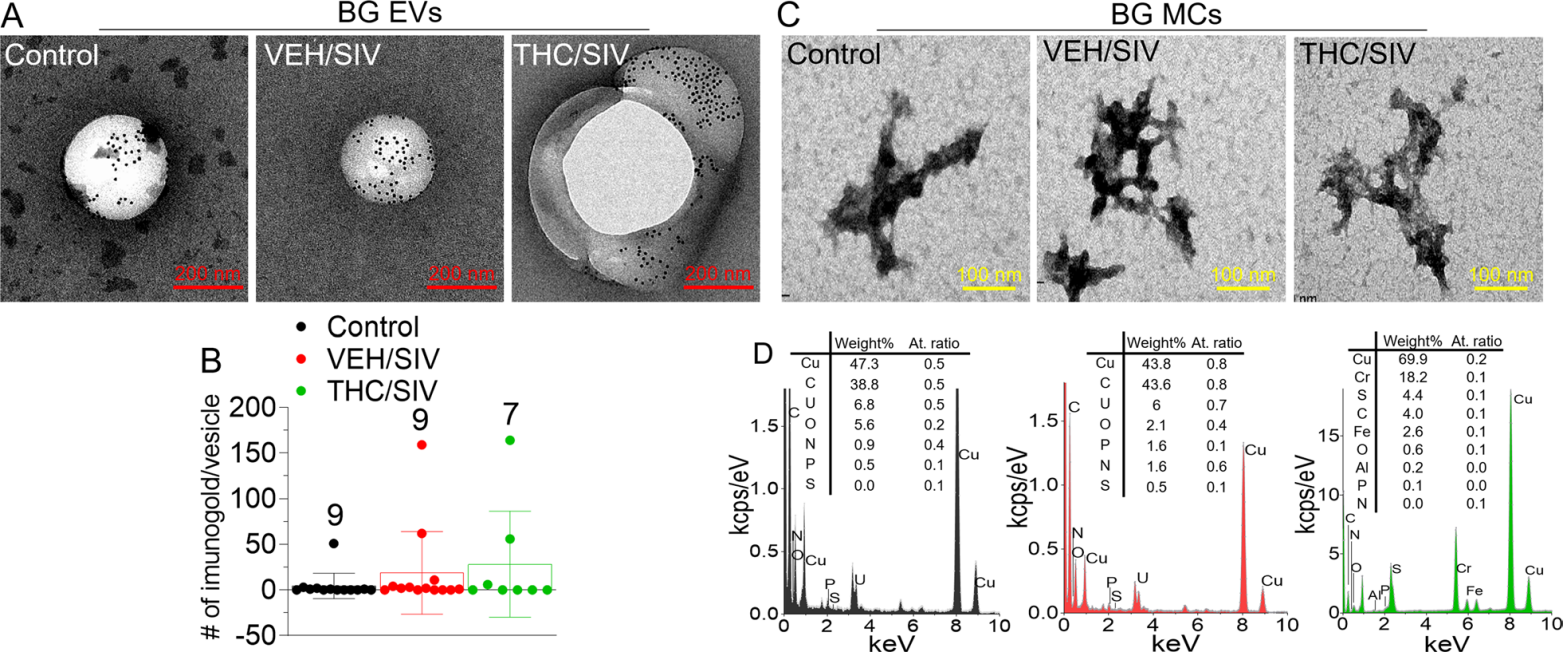
TEM–EDX elemental analysis of BG structures. **A** Representative TEM images of CD9-immunolabeled EVs from BG tissues, from the three RM groups. The black spots on the structures correspond to 6-nm-gold-nanoparticles-labeled goat anti-mouse secondary antibody. **B** Total of 25 TEM images were analyzed for size and counted for the immunogold label per vesicle and the means were reported per group. Error bar represent standard error of the mean. The number above the bars represent the number of vesicles analyzed per group. **C** Representative images of MC from BG tissues. Seen are dense, sharp-edged and membraneless structures of primary particle size of ~ 10–20 nm that aggregate into large structures up to ~ 500 nm. **D** Corresponding EDX spectra of the MC structures revealing presence of SPONCH (Sulphur, S; Phosphorus, P; Oxygen, O; Nitrogen, N; Carbon, C; and H, Hydrogen) elements, as a signature of biomolecules. Despite the strong signal of copper, Cu and chromium, Cr which constitute the main material of the specimen holder, and the fact that H is beyond the EDX detection limit and C may also be a specimen artifact, persistent traces of SPON, sulfur, phosphorus, oxygen, and nitrogen, were detected, supporting a ribonucleo-proteinaceous nature of the MC structures

TEM–EDX-IL analysis showed that BG-EVs (~ 100–300 nm) are enriched in CD9 (Fig. 2A, B). Whether other EV markers are present and to what extent is unknown and needs follow-up studies. Nevertheless, the purity of the BG-EVs preparation via PPLC, devoid of free proteins and other extracellular structures that may masquerade as EVs was demonstrated. We also analyzed the non-EV MC component of BG. Similar to seminal plasma [42], BG MCs exhibit dense, sharp-edged and membraneless structures with primary particle size of ~ 10–20 nm that aggregates into large structures up to ~ 500 nm (Fig. 2C). Trace elements detected in the EDX spectra of BG MCs confirmed the ribonucleo-proteinaceous nature of BG MCs (Fig. 2D). The analysis of the ultrastructure and chemical composition of EVs via this method provides rigor and is of the utmost importance for measuring the chemical composition of EVs and other particles. Moreover, the non-EV MCs are of interest to the EV field, because these structures often copurify with EVs and masquerade as EVs. Thus, showing that PPLC has the ability to separate EVs from non-EVs improved the rigor of our EV isolation technique.

### SIV downregulates proinflammatory miRNAs in BG-EVs and THC counteracts the effect of SIV 

**Fig. 3:**
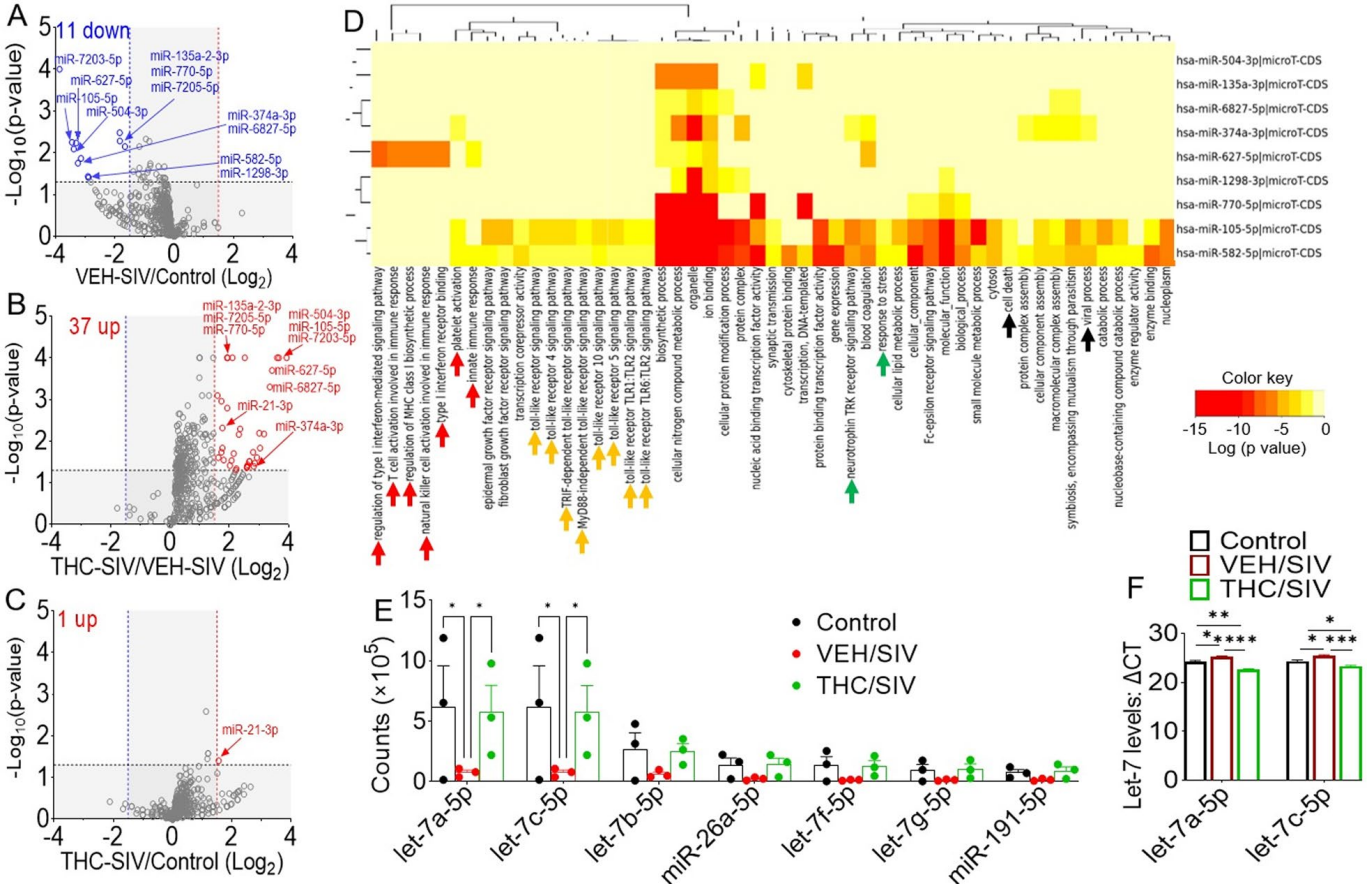
Changes in miRNA expression in BG-EVs from chronically SIV-infected rhesus macaques administered vehicle (VEH) or THC.** A–C** Volcano plots showing the relationship between fold-change and statistical significance of differentially expressed miRNAs in **A** VEH/SIV RMs relative to uninfected control, **B** THC/SIV relative to VEH/SIV RMs, and **C** THC/SIV relative to control RMs. The blue and red vertical lines correspond to 1.5-fold down and up, respectively, and the horizontal black lines represent *p* < *0.05*. The miRNAs of interest are denoted with arrows. **D** Heatmap of significant pathways predicted by DIANA-miRPath (v.3) of the human counterparts of the 11 SIV-downregulated miRs, showing inflammation and immune regulation pathway cluster (red arrows), a TLR signaling pathway cluster (orange arrows), in addition to Neurotrophin TRK receptor signaling pathway and response to stress (green arrows), as well as cell death and viral process (black arrows), among others. **E** Raw counts of selected neuroregulatory miRNAs, downregulated in the VEH/SIV group. **F** TaqMan PCR validation using mml-let-7a-5p and mml-let-7c-5p specific TaqMan microRNA stem-loop RT-qPCR assays. Statistical differences were assessed by a two-way ANOVA test with Tukey’s correction (*n* = 3). *p < 0.05

We analyzed miRNA content of BG-EVs using the Sequencing Metrics shown in Table 2. Relative to uninfected control BG-EVs, 11 significantly downregulated miRNAs were identified in BG-EVs of VEH/SIV (Fig. 3A, blue arrows). Remarkably, 37 miRNAs were significantly upregulated in THC/SIV, and these also included the 11 miRNAs downregulated in VEH/SIV BG (Fig. 3B, red arrows), along with miR-21 that was upregulated by THC (Fig. 3C, red arrow). DIANA pathway analysis of predicted miRNA target genes [63] identified various clusters, including inflammation/immune regulation (red arrow heads), TLR signaling (orange arrow heads), Neurotrophin TRK receptor signaling and response to stress (green arrow heads), cell death and viral process (black arrow heads) among the top-pathways (Fig. 3D). Moreover, BG-EVs from VEH/SIV RMs contained reduced levels of neuroregulatory miRNAs [64] (Fig. 3E), with significant differences in let-7a-5p and let-7c-5p. To confirm whether the level of let-7a-5p and let-7c-5p miRNA was reduced in BG-EVs from VEH/SIV RMs, we used mml-let-7a-5p and mml-let-7c-5p specific TaqMan microRNA stem-loop RT-qPCR assays. Both let-7a-5p and let-7c-5p were significantly lower in BG-EVs from VEH/SIV RMs than the levels in uninfected controls and THC/SIV-BG-EVs (Fig. 3F). These data reveal that the neuroprotective/anti-inflammatory factors induced by THC may be mediated by EV-associated miRNAs. Interestingly, BG viral loads were generally higher in VEH/SIV compared to THC/SIV RMs (Table 1), although the differences did not reach statistical significance (*p* = 0.100). The list of downregulated miRs in BG-EVs from VEH/SIV compared to uninfected control RMs is shown in Table 3, while Table 4 shows downregulated miRs in BG-EVs from THC/SIV compared to controls.

**Table 2:** Sequencing metrics* across each sample

Sample ID	Raw reads	Trimmed reads (minimum length 5)	Percentage reads (minimum length 5)	Trimmed reads (minimum length 15)	Percentage reads (minimum length 15)	Reads aligned to mmul genome	Percentage reads aligned to mmul genome	Reads aligned to mmul miRBase	Percentage reads aligned to mmul miRBase
IR97	15,040,626	14,916,991	99.2	14,726,019	97.9	5,801,187	39.39	197,985	1.34
IT18	10,553,653	10,383,011	98.4	10,123,258	95.9	8,449,318	83.46	3,437,862	33.96
GT18	8,502,723	8,407,642	98.9	8,250,430	97.0	7,014,323	85.02	2,224,050	26.96
IV95	12,202,389	11,909,194	97.6	11,402,173	93.4	8,043,460	70.54	180,656	1.58
JD66	13,541,260	13,405,955	99.0	13,232,393	97.7	11,158,775	84.33	593,001	4.48
JR36	9,531,380	9,304,961	97.6	8,964,346	94.1	7,063,324	78.79	353,166	3.94
JI45	11,677,881	11,421,702	97.8	10,918,406	93.5	8,837,853	80.94	3,226,625	29.55
JC85	10,290,459	10,095,165	98.1	9,675,224	94.0	6,639,058	68.62	752,874	7.78
IV90	9,073,363	8,899,837	98.1	8,538,395	94.1	6,826,220	79.95	2,065,222	24.19

*After adapters are trimmed, reads are filtered based on length (5 bp, 15 bp). Filtering reads shorter than 5 bp determines RNA degradation. 15 bp is the minimum length for meaningful alignments

**Table 3:** List of downregulated miRs in BG-EVs from VEH/SIV compared to control rhesus macaques

miRNA	Mean log read count (control)	*N* (control)	Mean log read count (VEH/SIV)	N (VEH/SIV)	Log2 fold change	− log (*P*-value)
mml-miR-7203-5p	0.1	3	1.465	3	− 3.87283	4
mml-miR-105-5p	0.1003	3	1.099	3	− 3.4538	2.251812
mml-miR-504-3p	0.1003	3	1.055	3	− 3.39485	2.091515
mml-miR-627-5p	0.1	3	0.9929	3	− 3.31165	2.236572
mml-miR-374a-3p	0.1003	3	0.954	3	− 3.24967	1.752027
mml-miR-6827-5p	0.1	3	0.8882	3	− 3.15088	1.866461
mml-miR-1298-3p	0.1	3	0.7518	3	− 2.91035	1.434152
mml-miR-582-5p	0.1	3	0.7426	3	− 2.89259	1.407823
mml-miR-135a-2-3p	0.4101	3	1.469	3	− 1.84079	2.481486
mml-miR-770-5p	0.392	3	1.398	3	− 1.83444	2.283997

**Table 4:** List of upregulated miRs in BG-EVs from THC/SIV compared to VEH/SIV rhesus macaques

miRNA	Mean log read count (VEH/SIV)	N (VEH/SIV)	Mean log read count (THC/SIV)	N (THC/SIV)	Log2 fold change	− log (*p*-value)
mml-miR-7203-5p	1.533	3	0.1	3	3.938286	4
mml-miR-504-3p	1.283	3	0.1003	3	3.677128	4
mml-miR-105-5p	1.233	3	0.1003	3	3.619779	4
mml-miR-627-5p	1.092	3	0.1	3	3.448901	3.69897
mml-miR-6827-5p	1.033	3	0.1	3	3.368768	3.30103
mml-miR-191-3p	0.9053	3	0.1003	3	3.174074	2.173925
mml-miR-107-5p	0.8257	3	0.1003	3	3.041296	1.835647
mml-miR-323b-5p	0.8105	3	0.1	3	3.018812	2.19382
mml-miR-190b	0.767	3	0.1003	3	2.934905	1.605548
mml-miR-374a-5p	0.731	3	0.1003	3	2.86555	1.47237
mml-miR-374a-3p	0.7101	3	0.1003	3	2.823701	1.396856
mml-miR-16-1-3p	0.7001	3	0.1	3	2.807561	1.735182
mml-miR-542-5p	0.6414	3	0.1	3	2.681224	1.511449
mml-miR-892c-5p	0.6414	3	0.1	3	2.681224	1.511449
mml-miR-448	0.6191	3	0.1	3	2.630172	1.430626
mml-let-7g-3p	0.6065	3	0.1	3	2.600508	1.386158
mml-miR-487b-5p	0.6065	3	0.1	3	2.600508	1.386158
mml-miR-379-3p	0.6021	3	0.1	3	2.590003	1.369572
mml-miR-206	1.491	3	0.2594	3	2.52303	4
mml-miR-30c-1-3p	1.033	3	0.2007	3	2.363728	2.29243
mml-miR-1296-5p	1.003	3	0.2007	3	2.321209	2.161151
mml-miR-4766-5p	0.7518	3	0.159	3	2.241322	1.337242
mml-miR-532-3p	0.7418	3	0.159	3	2.222004	1.302771
mml-miR-7184-3p	0.8925	3	0.2007	3	2.152812	1.701147
mml-miR-770-5p	1.689	3	0.392	3	2.107244	4
mml-miR-455-5p	0.8488	3	0.2007	3	2.080384	1.536107
mml-miR-1255a-5p	0.8157	3	0.2007	3	2.022998	1.415669
mml-miR-135a-2-3p	1.58	3	0.4101	3	1.945877	4
mml-miR-490-3p	1.27	3	0.3333	3	1.929935	2.79588
mml-miR-7205-5p	1.702	3	0.4475	3	1.927271	4
mml-miR-128a-5p	0.9108	3	0.2594	3	1.811956	1.548214
mml-miR-21-3p	1.184	3	0.3471	3	1.770246	2.318759
mml-miR-194-5p	1.026	3	0.301	3	1.769195	1.835647
mml-miR-874-5p	1.001	3	0.301	3	1.733607	1.735182
mml-miR-184	1.39	3	0.4184	3	1.73213	2.958607
mml-miR-320c	0.9847	3	0.3181	3	1.630204	1.605548
mml-miR-137-5p	1.492	3	0.4924	3	1.599345	3.09691

To confirm whether the level of let-7a-5p and let-7c-5p miRNA was reduced in BG-EVs from VEH/SIV RMs, we used mml-let-7a-5p and mml-let-7c-5p specific TaqMan microRNA assays. Both let-7a-5p and let-7c-5p were significantly lower in BG-EVs from VEH/SIV RMs compared to the levels in uninfected controls and SIV/THC-BG-EVs (Fig. 3F). These data reveal that the neuroprotective/anti-inflammatory factors induced by THC may be mediated by EV-associated miRNAs. Interestingly, BG viral loads were generally higher in VEH/SIV compared to THC/SIV RMs (Table 1), although the differences did not reach statistical significance (*p* = 0.100). The list of downregulated miRs in BG-EVs from VEH/SIV compared to control RMs is shown in Table 3, while Table 4 presents downregulated miRs in BG-EVs from THC/SIV compared to controls.

### Primary astrocytes internalize BG-EVs

**Fig. 4:**
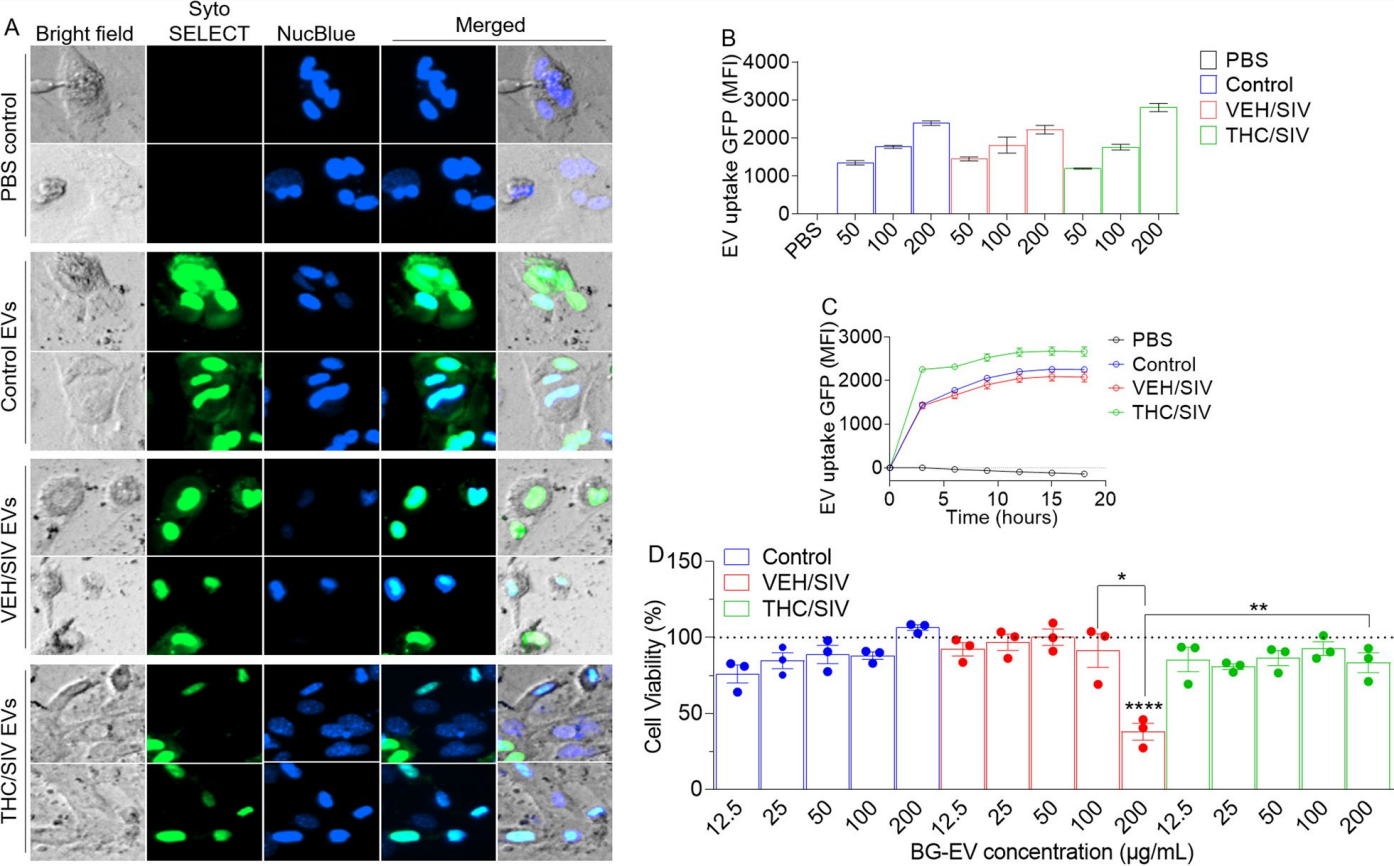
BG-EVs are taken up by astrocytes: we incubated 10,000 astrocytes from *CX3CR1*^+/GFP^ mice with increasing concentrations (0, 12.5, 25, 50, 100 and 200 µg/mL) of SytoSELECT labeled BG-EVs in the presence of NucBlue (a live cell stain, 30 µL/mL) and seeded in a glass-bottom 96 well plate 24-h prior to experiment. Kinetics imaging (every 3 h) was recorded over the course of 18 h using automated Lionheart FX software [18] (Biotek) after which total GFP (ex/em) and Nucblue (ex/em) intensity were recorded using a plate reader (Synergy H1 Biotek). Astrocytes were assessed for viability using Cell Titer Glow assay (Promega). **A** Representative 10 × images at 18 h timepoint after addition of BG-EVs. **B** Total gfp and nucblue intensity as measured by the plate reader. **E** Viability of astrocytes treated with BG-EVs. Ordinary one-way ANOVA multiple comparison test (Tukey’s test) was used to assess statistical differences. *p < 0.05; **p < 0.01; ***p < 0.001; ****p < 0.0001; ns, non-significant. Error bars represent Standard Deviation

To determine the biological significance of EVs and their cargo isolated from the different treatments groups, we incubated BG-EVs isolated from uninfected controls, VEH/SIV and THC/SIV RMs with in vitro cultured primary mouse astrocytes. The internalization of the labeled EVs by astrocytes revealed a green signal (Fig. 4A) that increased with increasing concentration (Fig. 4B) and extended time (Fig. 4C). Treating cells with increasing concentrations of EVs for 18 h showed that EVs from uninfected control and THC/SIV RMs at all concentrations did not alter astrocyte viability. However, 200 µg/mL of BG-EVs from VEV/SIV RMs reduced astrocyte viability but the same concentration of EVs from THC/SIV BG had no impact on viability (Fig. 4D). These functional data suggest that high amounts of EVs from SIV-infected animals may have the potential to compromise astrocyte survival. The reversal of SIV EV-induced decrease in cell viability by THC/SIV EVs suggests that EVs from HIV/SIV-infected brain (BG) cells contain factors/molecules that may induce oxidative stress and impair cellular metabolism that are absent or excluded in EVs isolated from BG of THC/SIV macaques. How EVs interact with astrocytes is unknown, although such interaction may be receptor mediated or independent of receptor. It is estimated that each BG-EV weighs 200–800 fg on average (Fig. 1H, bottom). A single treatment of 10,000 cells with 100 μg EVs corresponds to a cell:EV ratio of 1:12,000–1:4000. With this amount of EVs, and after a period of 18–24 h, most astrocytes (> 90%) internalized EVs. Despite this, it is still unclear why high concentration of BG-EVs from THC/SIV BG counteracted the effects of BG-EVs from VEV/SIV macaques. Nonetheless, cannabinoid receptors are present in the CNS, including neocortex, hippocampus, basal ganglia, cerebellum, and brainstem, and data from the Human Protein Atlas show that the BG tissues and astrocytes express varying levels of cannabinoid receptors (Table 5).

**Table 5:** Cannabinoid receptors RNA expression levels (from the Human Protein Atlas: proteinatlas.org)

Gene name	Gene description	Consensus nTPM*					
		Basal ganglia	Microglial cells	Inhibitory neurons	Excitatory neurons	Astrocytes	Oligodendrocytes
CNR1	Cannabinoid receptor 1	25.1	5.6	200.3	44.6	3.6	3.2
CNR2	Cannabinoid receptor 2	0.1	0.0	0.4	0.4	0.3	0.5
GPR55	G protein-coupled receptor 55	4.6	1.0	0.1	2.3	0.3	0.1
GPR18	G protein-coupled receptor 18	0.3	0.0	0.4	0.4	0.3	0.1

*nTPM: normalized transcript per million

### BG-EVs activate primary astrocytes

**Fig. 5:**
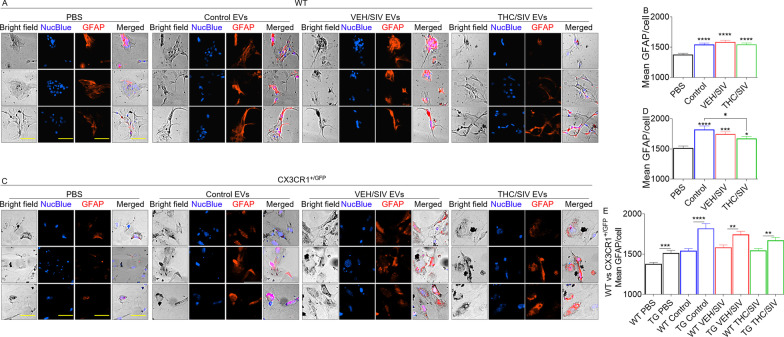
BG-EVs elevate Glial Fibrillary Acidic Protein (GFAP) levels: 10,000 primary mouse astrocytes per well were seeded in a glass-bottom 96 well plate 24-h prior to experiment. Cells were treated with vehicle PBS or with 100 µg/mL pooled BG-EVs (*n* = 4, 25 µg/pool from each group) for 24 h, in triplicate wells and 9 field of views. Cells were then fixed, immuno-stained for GFAP, and imaged using an automated scope (Lionheart FX, Biotek). **A, C** Representative 10 × images for **A** WT and **C** CX3CR1 + GFP + astrocytes. Scale bar = 50 µm. **B, D** Mean fluorescence intensity (MFI) of GFAP calculated using single-cell analysis for **B** WT and **D** CX3CR1 + GFP + astrocytes. Ordinary one-way ANOVA multiple comparison test (Dunnett’s test) was used to assess statistical differences in **B** and **D**. *p < 0.05; ***p < 0.001; ****p < 0.0001. Error bars represent S.E.M. of 600–1000 cells per treatment. **E** Graph depicting genotype-dependent differential response to the treatments between WT and CX3CR1 + GFP + astrocytes. Unpaired *T* test with Welch’s correction was used to assess statistical differences. **p < 0.01; ***p < 0.001; ****p < 0.0001. Error bars represent S.E.M. of 600–1000 cells per treatment

Reactive astrocytes are polarized by insults toward neurotoxic A1 (inflammatory/harmful) or neuroprotective A2 (protective) phenotypes, with A1 phenotypes being linked to neurodegenerative diseases and aging [65]. In our study, all the EVs irrespective of background (uninfected controls, VEH/SIV, THC/SIV) upregulated the expression of GFAP, a marker of reactive/activated astrocytes (Fig. 5A-–D). WT and CX3CR1^+/GFP^ astrocytes treated with uninfected control, VEH/SIV, and THC/SIV EVs significantly elevated GFAP (Fig. 5B, D). However, in CX3CR1^+/GFP^ cells, THC/SIV EVs significantly suppressed GFAP levels compared to uninfected control EVs (Fig. 5D). While BG-EVs elevated GFAP expression in WT and CX3CR1^+/GFP^ cells, CX3CR1^+/GFP^ cells are more activated as shown by significantly higher GFAP levels (Fig. 5E).

### BG-EVs alter the transcriptome of primary astrocytes

**Fig. 6:**
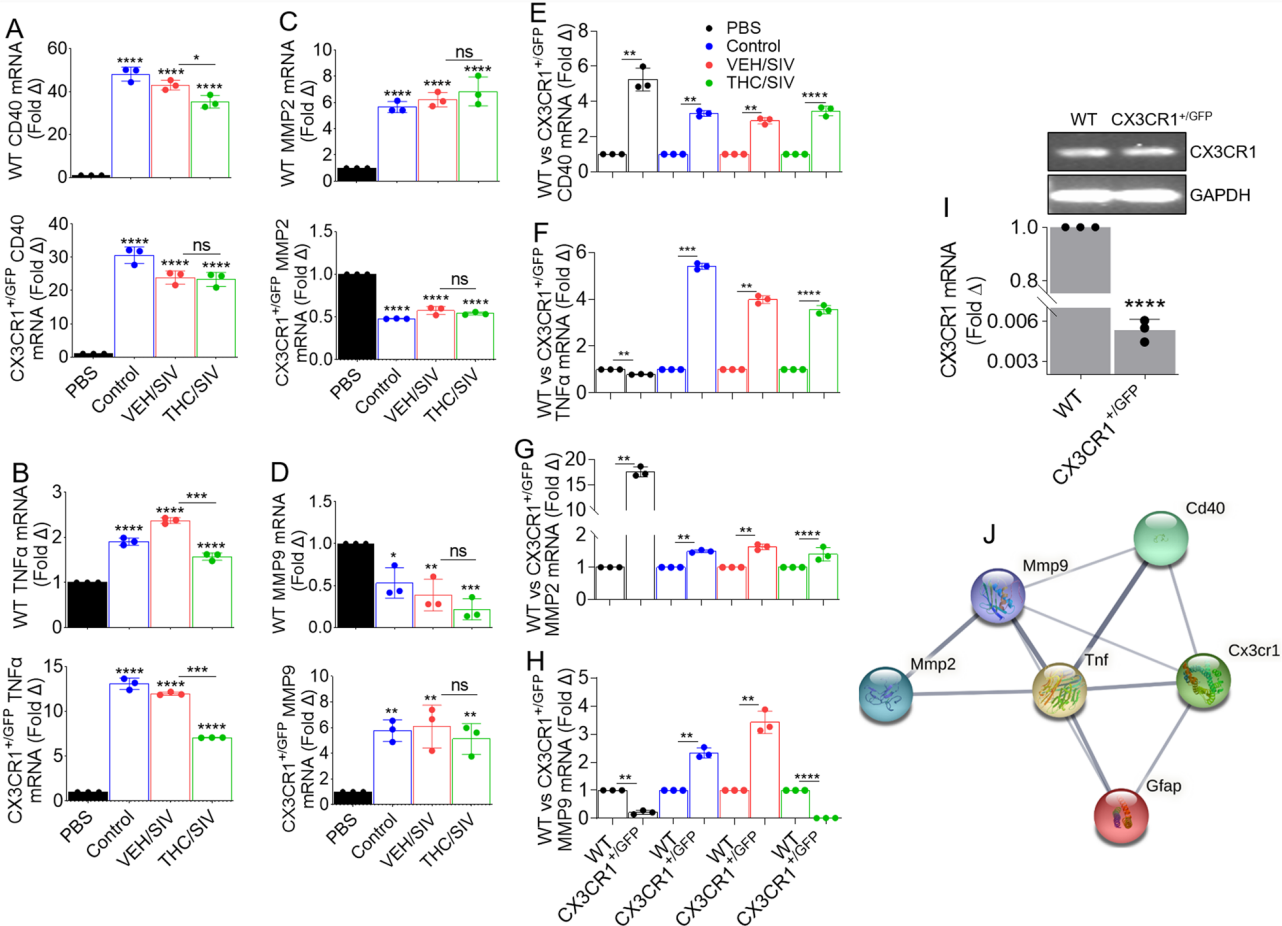
BG-EVs mediate distinct changes in the transcriptome of astrocytes: 200,000 primary mouse astrocytes per well were seeded in a 12 well-plate 24-h prior to experiment. Cells were treated with vehicle PBS or with 100 µg/mL pooled BG-EVs (*n* = 4, 25 µg/pool from each group) and incubated for 24 h. Cellular RNA was extracted, and gene expression was assessed by RT-qPCR. **A–D** Differential analysis of **A** CD40, **B** TNFα, **C** MMP2, and **D** MMP9. Top graph is WT and bottom graph is CX3CR1 + GFP + (TG) astrocytes. **E–H** Graphs depicting the differential response to the treatments between WT and CX3CR1 + GFP + (TG) astrocytes. Ordinary one-way ANOVA multiple comparison test (Dunnett’s test) was used to assess statistical differences in **A**–**D**. Unpaired *T* test with Welch’s correction was used to assess statistical differences between SIV and THC/SIV groups in panels A–H. Error bars represent S.E.M. *p < 0.05; **p < 0.01; ***p < 0.001; ****p < 0.0001; ns, not significant. **I** The levels of untreated astrocyte CX3CR1 mRNA expression relative to normalization against GAPDH. The RT-qPCR products representative of mRNA levels were normalized to GAPDH signal and shown on the bar, while the amplicons separated on agarose gel is the inset. **J** Protein–protein interaction (PPI) network of the altered genes in astrocytes

Primers shown in Table 6 were used to quantify the mRNA profile of a select list of cellular activation genes in astrocytes. EVs from all groups increased mRNA expression of CD40 (TNF-receptor superfamily) in WT and CXCR1^+/GFP^ astrocytes (Fig. 6A). EVs also elevated TNFα mRNA in WT and CX3CR1^+/GFP^ astrocytes (Fig. 6B). Interestingly, THC/SIV EVs significantly reduced CD40 mRNA in WT but not CX3CR1^+/GFP^ astrocytes (Fig. 6A) but suppressed TNFα mRNA in both WT and CX3CR1^+/GFP^ astrocytes (Fig. 6B).

**Table 6:** Mouse primer* sequences used in this study

Gene	Forward sequence (5′→3′)	Reverse sequence (5′→3′)
GAPDH	CCCCTTCATTGACCTCAACTACA	CGCTCCTGGAGGATGGTGAT
CD40	TGAGGATAAGAACTTGGAGGTC	CCGGGACTTTAAACCACAGA
TNFα	CACCACCATCAAGGACTC	AGGTCTGAAGGTAGGAAGG
MMP-2	TCAAGGACCGGTTTATTTGG	GCGAAGAACACAGCCTTCTC
MMP-9	CAGCCGACTTTTGTGXXCTT	GCTTCTCTCCCATCATCTGG
CX3CR1	AGGACACAGCCAGACAAG	TCAGGGGAGAAAGCAAG

Because HIV regulates MMP expression in astrocytes [66], we assessed the regulation of astrocyte MMP levels in response to BG-EV treatment. The EVs irrespective of background elevated MMP2 mRNA in WT astrocytes but suppressed MMP2 mRNA in CX3CR1^+/GFP^ cells (Fig. 6C). In contrast to MMP2, EVs irrespective of background suppressed MMP9 mRNA in WT astrocytes while upregulating MMP9 mRNA in CX3CR1^+/GFP^ astrocytes (Fig. 6D). Compared to WT, EVs more significantly elevated CD40 (Fig. 6E), TNFα (Fig. 6F), MMP2 (Fig. 6G), and MMP9 (Fig. 6H) mRNA in CX3CR1^+/GFP^ astrocytes. The exceptions were suppression of TNFα by PBS (Fig. 6F) and suppression of MMP9 by PBS and THC/SIV EVs in CX3CR1^+/GFP^ cells (Fig. 6H). These data show that EVs regulate astrocyte CD40, MMP2, and MMP9 in a CX3CR1-dependent manner, since WT astrocytes express significantly higher CX3CR1 mRNA compared to CX3CR1^+/GFP^ astrocytes (Fig. 6I). Although some reports indicate that astrocytes do not express CX3CR1 [67, 68], prior studies showed that astrocytes express CX3CR1 and GFAP [69], both in vitro and in vivo and during brain development or neurological insult [70]. Moreover, primary simian astrocytes also express CX3CR1 mRNA [71]. Functional enrichment analysis (using String) revealed the interactome of GFAP, CD40, TNFα, MMP2, and MMP9 (Fig. 6J). GFAP, CD40, TNFα, MMP2, and MMP9 are enriched in numerous gene ontology (GO) biological processes (Table 7) and KEGG pathways (Table 8). Of particular interest was the enrichment of positive regulation of ikappaB phosphorylation, regulation of chronic inflammatory response, regulation of immunoglobulin secretion, positive regulation of glial cell proliferation, and leukocyte tethering/rolling (Table 7). We also found the enrichment of asthma, bladder cancer, allograft rejection, malaria, and viral protein interaction with cytokine and cytokine receptor (Table 8) to be interesting. These data are noteworthy, because increased TNFα and GFAP expression are found in the brains of patients with certain CNS disorders [72], and exogenous TNFα suppressed GFAP expression in cultured astrocytes [73].

**Table 7:** Top-10 GO biological process, depicting functional enrichment in the network shown in Fig. 6J

#Term ID	Term description	Observed gene count	Background gene count	Strength	False discovery rate
GO:1903721	Positive regulation of i-kappab phosphorylation	2	6	3.09	0.0019
GO:0002676	Regulation of chronic inflammatory response	2	11	2.82	0.0036
GO:0051023	Regulation of immunoglobulin secretion	2	19	2.59	0.0051
GO:0060252	Positive regulation of glial cell proliferation	2	20	2.57	0.0054
GO:0050901	Leukocyte tethering or rolling	2	21	2.54	0.0055
GO:1904707	Positive regulation of vascular-associated smooth muscle cell proliferation	3	35	2.5	0.00082
GO:0030574	Collagen catabolic process	2	30	2.39	0.0085
GO:0031102	Neuron projection regeneration	2	32	2.36	0.0094
GO:0001774	Microglial cell activation	2	33	2.35	0.0097
GO:2001240	Negative regulation of extrinsic apoptotic signaling pathway in absence of ligand	2	33	2.35	0.0097

**Table 8:** Gop-10 KEGG pathways, depicting functional enrichment in the network shown in Fig. 6J

#Term ID	Term description	Observed gene count	Background gene count	Strength	False discovery rate
mmu05310	Asthma	2	24	2.49	0.0034
mmu05219	Bladder cancer	2	40	2.26	0.0048
mmu05330	Allograft rejection	2	50	2.17	0.0062
mmu05144	Malaria	2	53	2.14	0.0062
mmu04061	Viral protein interaction with cytokine and cytokine receptor	2	87	1.93	0.0136
mmu01522	Endocrine resistance	2	91	1.91	0.0136
mmu04657	IL-17 signaling pathway	2	90	1.91	0.0136
mmu05322	Systemic lupus erythematosus	2	93	1.9	0.0136
mmu05418	Fluid shear stress and atherosclerosis	3	141	1.89	0.0024
mmu04620	Toll-like receptor signaling pathway	2	98	1.88	0.0136

## Discussion

HIV invades the brain within 2 weeks after infection [74, 75], infecting resident CNS cells, including microglia and astrocytes [76, 77]. Glial cells maintain brain homeostasis [78] via scavenging for excess toxic neurotransmitters, maintaining BBB integrity, regulation of immune activation/inflammation, and release of neurotrophic factors. Moreover, activation of glial cells contributes to neuropathology induced by mitochondrial toxins [79]. HIV and its proteins (Tat and gp120) have been implicated in mediating astrocyte toxicity [80, 81]. Although astrocytes make up about 40% of the total CNS cell population, their exact function in HIV-induced neuroinflammation remains unclear.

Our data using the SIV-infected RM model exposed to chronic THC treatment, along with WT and CX3CR1^+/GFP^ astrocytes, revealed that BG contains EVs with previously unrecognized functions. There was no significant difference in BG-EV size distributions, concentrations, protein content, and zeta potential (Fig. 1). Similarly, we detected no difference in the structure of EVs (Fig. 2) from BG samples of uninfected control, VEH/SIV, and THC/SIV RMs. However, significant differences were observed in the BG-EV miRNA profile (Fig. 3). The cargo composition and functions of the BG-EVs were modulated by HIV/SIV infection. BG-EVs from these animals exhibited a proinflammatory profile and induced an activated/proinflammatory state in primary CNS astrocytes. Strikingly, long-term low dose THC treatment of SIV-infected RMs selectively counteracted the generalized proinflammatory nature of SIV BG-EVs. Specifically, both mml-let-7a-5p and mml-let-7c-5p were significantly lower in BG-EVs from VEH/SIV RMs than the levels in uninfected controls and THC/SIV-BG-EVs (Fig. 3F). Interestingly, the let-7 family members which have identical seed sequence [82] are abundantly expressed in the brain, glial progenitor cells, astrocytes [83] and they exhibit high cross-species sequence conservation [84–86]. The let-7 family of miRNAs is involved in regulating CNS inflammation and neurological outcomes. Studies have linked let-7a-5p and let-7c-5p overexpression to the suppression of TNFα expression [87, 88], while let-7c-5p improved neurological outcomes in a murine model of traumatic brain injury by suppressing neuroinflammation [89]. With respect to HIV, Swaminathan et al., showed significant down-regulation of Let-7 family of miRNAs in patients with chronic HIV infection compared to healthy controls [90], while Zhang et al. showed that HIV infection resulted in suppression of the let-7i/IL-2 axis leading to cell death [91]. Aside from HIV, let-7d-5p, let-7a, let-7c, and miR-122-5p decreased over time in agreement with the progression of liver fibrosis in hepatitis C-infected people [92]. In the context of EVs, let-7a has been shown to regulate EV secretion and mitochondrial oxidative phosphorylation [93]. Furthermore, EV-associated let-7a-5p and let-7c-5p levels were significantly reduced in liver cirrhosis patients and let-7a-5p levels significantly correlated with hepatic fibrosis markers and could predict hepatic cirrhosis more accurately than other markers of hepatic fibrosis [92].

It is evident that EVs can be used as biomarkers for specific conditions, as suggested for neuron-derived EVs from PLWH bearing biomarkers of cognitive impairment [94]. EVs are also prospective carriers of drugs and other exogenous compounds with the potential to regulate neuropathogenesis [95]. Using the SIV-infected RM model, we detected several miRNAs that regulate inflammation/immune regulation, TLR signaling, Neurotrophin TRK receptor signaling, cell death/response to stress. Neurotrophins, including brain-derived neurotrophic factors (BDNF) are a family of closely related proteins identified to control many aspects of survival, development, and functions of neurons. Continued presence of neurotrophins is essential as it controls synaptic function and plasticity, sustain neuronal survival, morphology, and differentiation, in addition to other roles outside the nervous system [96]. Furthermore, HIV suppresses BDNF expression and reduces BDNF activity, resulting in neurodegeneration in infected individuals [97, 98]. Remarkably, long term low dose THC administration led to significant upregulation of all the SIV-downregulated miRNAs. THC also upregulated 26 miRNAs and some neuromodulatory miRNAs, including the let-7 family members [64] that regulate biological processes, such as apoptosis [99, 100], immune system modulation [101, 102], TLR7 activation [103], axon guidance [104], and BBB permeability [105]. In addition, let-7c has been shown to promote polarization of macrophages from M1 to M2 phenotype [102]. These neuromodulatory miRNAs were either downregulated or were unchanged in the VEH/SIV group. The various pathways altered by SIV or THC have been implicated in neuroinflammation. These observations indicate that the changes SIV and/or THC imprinted on the brain, manifest in BG-EVs, which may then serve as a conduit for dissemination of miRNAs to CNS cells. It is also possible that BG-EVs may spread their miRNA cargos to distant sites in the periphery via cell-to-cell transfer.

Aside from serving as biomarkers, EVs mediate intercellular communication, both within and across species. The cross-species efficacy of EVs and their cargo has been established by our group and others. For example, human semen-derived EVs delivered human Apobec3g and Apobec3f gene products to mice in vivo [106]. Moreover, EVs derived from human BMD2a cells were incorporated within mice brains and the EVs mediated permeability of mouse brain blood vessels [107]. If EVs mediate intercellular communication via their cargo, and EVs from one species can function in another species, cross-species transfer of miRNA may be likely, especially since miRNAs are conserved throughout bilaterian evolution [108]. In our study, mouse astrocytes tolerated up to 100 μg of rhesus macaque BG-EVs. However, increasing BG-EV concentration beyond 100 µg showed that the tolerance of astrocytes was dependent on the origin (uninfected control, VEH/SIV, THC/SIV) and concentration (50, 100, 200 µg) of the EVs. The viability of astrocytes remained unchanged and comparable to PBS treated cells and tolerated up to 200 µg EV concentration. However, viability of astrocytes significantly declined in the presence of 200 µg of VEH/SIV EVs. THC/SIV EVs prevented VEH/SIV EV-mediated decline in astrocyte viability.

In the steady state, astrocytes express GFAP. However, the expression of GFAP is increased during activation [109]. In our study, VEH/SIV EVs significantly increased the level of astrocyte GFAP (Fig. 5). The conversion of GFAP^low^ astrocytes into GFAP^high^ astrocytes which occurred via interaction with EVs is indicative of their potential to activate these cells. Activated astrocytes, and other brain resident cells are key contributors to HIV-induced neuroinflammation. These cells release neurotoxic factors and inflammatory mediators such as TNFα [110] that may lead to deleterious consequences, including neurotoxicity. TNFα is mitogenic to astrocytes and increased levels of TNFα are associated with elevated GFAP expression [111]. Astrocytes promote chronic inflammation and progressive neurodegeneration via overexpression of TNFα [112]. Increased production of TNFα is linked to HIV-induced immunological abnormalities [113–115] and astrocyte apoptosis, a major feature of cellular injury in HAND [116]. In the CNS, CD40 is expressed by astrocytes and its interaction with CD40L on other resident CNS cells such as T, monocytic, natural killer, and mast cells mediates intracellular signaling events that promote the production of proinflammatory factors and neurotoxins [117]. The reprogramming of astrocytes from CD40/TNFα^low^ to CD40/TNFα^high^ by EVs is suggestive of the proinflammatory nature of VEH/SIV BG-EVs. Similarly, the conversion of CD40/TNFα^high^ to CD40/TNFα^intermediate^ by THC/SIV EVs is suggestive of the potential anti-inflammatory nature of THC/SIV EVs. These observations are significant, because the hallmarks of HAND include widespread microglial activation, accompanied by reactive astrogliosis and their secretory products, including cytokines and chemokines [118, 119].

In addition to TNFα, other inflammatory mediators were induced by BG-EVs. MMP2 and MMP9 mRNA were variably altered in astrocytes by all EVs in a CX3CR1-dependent manner. In some cases, THC/SIV EVs counteracted SIV EV-induced effects on MMP2 and MMP9 mRNA. These observations are intriguing, because under pathological conditions, dysregulated expression of MMPs induces inflammation and promotes progression of neurodegenerative diseases [120]. MMP2 and MMP9 are ECM-degrading enzymes involved in inflammation and tissue remodeling. Through their induction of soluble TNFα and proteolytic activity on the ECM, MMPs may promote brain injury. It is likely that astrocyte activation by EVs may trigger the expression of inflammatory mediators (TNFα, MMP2, and MMP9) as observed in the present study. Furthermore, MMPs are thought to be involved in the pathogenesis of HAND and other neurodegenerative disorders via degradation of ECM and compromising the BBB [121–123]. In addition, MMP2 and MMP9 are present in the CSF, plasma, and brain tissue of HIV patients [122]. The suppressive effect of THC/SIV EVs on inflammatory mediators show the potential of THC/SIV EVs to ameliorate the effect of pathological VEH/SIV EVs.

With regard to the role of CX3CR1 in HIV infection, the ligand of CX3CR1, CX3CL1, also known as Fractalkine (FKN) is increased in the CSF of HIV-infected individuals who exhibit neurocognitive impairment [124, 125]. However, exogenous FKN has been shown to protect cultured neurons from neurotoxicity induced by Tat or Tat + morphine-induced dendritic losses [126, 127]. It is worth mentioning that CX3CR1 isoforms produced by alternative splicing may function as fusion co-receptors for HIV envelope protein [128], although the significance of CX3CR1 among other HIV co-receptors for HIV entry is still not clear. Nonetheless, HIV-infected individuals homozygous for CX3CR1-I249 M280 (that affects two amino acids—isoleucine-249 and methionine-280) exhibit a more rapid progression to AIDS [129], perhaps due to reduced FKN binding. Our findings, together with the literature evidence, suggest a possible involvement of CX3CR1 mediated response to BG-EV alteration of astrocyte gene expression and function.

The ability of low dose THC, which is also prescribed [FDA-approved synthetic THC (Marinol)] as an appetite stimulant in PLWH [130–134] to reprogram BG-EVs and affect their functions is significant. Chronic cannabis use may slow disease progression, prolong survival, reduce viral load, and attenuate infection-induced inflammation/immune activation in SIV-infected RMs [7, 55–57, 135–138] and ART-treated PLWH [139, 140]. The effect of THC is systemic―affecting many organs. As a result, THC and other cannabinoids are recommended for the treatment of digestive disorders [141–146] and FDA approved their use for clinical management of wasting and appetite stimulation, in PLWH [130, 131, 133, 134, 147].

It is also evident in the comparative analysis of plasma and BG viral loads (Table 1), that BG viral loads were generally lower in the THC/SIV group compared to the VEH/SIV group, although not statistically significant. The lack of significance in viral load between the two groups may be due to the limited number (*n* = 3/group) of study subjects used. Thus, studies with increased sample size are warranted to assess whether THC can reduce CNS viral load.

## Conclusions

In summary, the findings of this study suggest that HIV/SIV infection reprograms the BG leading to the release of pathogenic EVs that may potentially promote CNS inflammation and toxicity. However, cannabinoid mediated modulation of EV cargo composition as shown in this study maybe a mechanism for the regulation of HIV/SIV-induced changes. This is significant, because exploration of the potential of THC EVs in a preclinical animal model may be logical to investigate whether the clinical advantages of THC EVs will result in beneficial outcomes. The findings of this study also pave the way for investigation into the effects of the combined administration of THC:CBD [1:1 or 1:3 ratio] on neuroinflammation and their effects on BG-EV composition and function. The implication of our findings goes beyond HIV-induced inflammation. Glia cells (microglia and astrocytes) are involved in the pathogenesis of pain [148]. Activated/reactive astrocytes play a role in neuropathic pain [149, 150], inflammatory pain [151, 152], as well as bone cancer pain [153]. Activated astrocytes are also involved in Parkinson’s disease, spinal cord injury [154, 155], and traumatic brain injury [156]. In line with their role in the pathogenesis of pain, studies are warranted to assess the effect of CNS EVs in mediating the development and maintenance of pain.

## Supplementary Information

**Additional file 1:** Schematic of BE-EV isolation process.

**Additional file 2:** Raw counts of BE-EV miRNA.

## Data Availability

The sRNA-Seq data sets are included within the article and its additional files.

## Abbreviations

BG: Basal ganglia
EV: Extracellular vesicles
CBD: Cannabidiol
CNS: Central nervous system
THC: Delta-9-tetrahydrocannabinol
MC: Membraneless condensates
VEH: Vehicle
