# Supplementary figures and images for: Chronic delta-9-tetrahydrocannabinol (THC) treatment counteracts SIV-induced modulation of proinflammatory microRNA cargo in basal ganglia-derived extracellular vesicles
Source: J Neuroinflammation. 2022 Sep 12;19:225. doi: 10.1186/s12974-022-02586-9 (PMC9469539; doi:10.1186/s12974-022-02586-9)

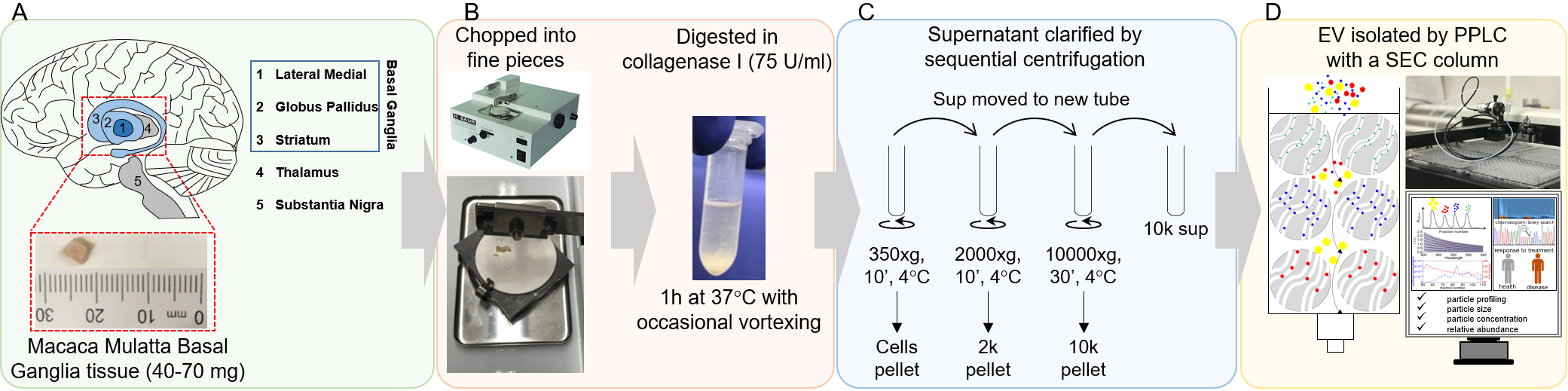

Supplement: Supplementary file 1 — Additional file 1: Schematic of BE-EV isolation process. [file 12974_2022_2586_MOESM1_ESM.tiff]
